# ZnO-Doped Metal-Organic Frameworks Nanoparticles: Antibacterial Activity and Mechanisms

**DOI:** 10.3390/ijms241512238

**Published:** 2023-07-31

**Authors:** Huiying Zheng, Biying Zhong, Qiaowen Wang, Xi Li, Jiehan Chen, Li Liu, Tiantian Liu

**Affiliations:** School of Public Health, Guangdong Pharmaceutical University, Guangzhou 510310, China; 1700202240@gdpu.edu.cn (H.Z.); zby_1232393@163.com (B.Z.); wqw_hhh@163.com (Q.W.); lixi8813@163.com (X.L.); chenjie.han@163.com (J.C.); pupuliu919@163.com (L.L.)

**Keywords:** metal-organic frameworks, MIL-101(Fe), ZnO, antibacterial activity, antibacterial mechanism

## Abstract

Metal-Organic Frameworks (MOFs) offer new ideas for the design of antibacterial materials because of their antibacterial properties, high porosity and specific surface area, low toxicity and good biocompatibility compared with other nanomaterials. Herein, a novel antimicrobial nanomaterial, MIL-101(Fe)@ZnO, has been synthesized by hydrothermal synthesis and characterized by FTIR, UV-vis, ICP-OES, XRD, SEM, EDS and BET to show that the zinc ions are doped into the crystal lattice of MIL-101(Fe) to form a Fe-Zn bimetallic structure. MIL-101(Fe)@ZnO was found to be effective against a wide range of antibacterial materials including *Staphylococcus aureus*, *Escherichia coli*, *Pseudomonas aeruginosa*, *Acinetobacter baumannii*, *Acinetobacter junii* and *Staphylococcus epidermidis*. It has a significant antibacterial effect, weak cytotoxicity, high safety performance and good biocompatibility. Meanwhile, MIL-101(Fe)@ZnO was able to achieve antibacterial effects by causing cells to produce ROS, disrupting the cell membrane structure, and causing protein leakage and lipid preoxidation mechanisms. In conclusion, MIL-101(Fe)@ZnO is an easy-to-prepare antimicrobial nanomaterial with broad-spectrum bactericidal activity and low toxicity.

## 1. Introduction

Currently, about four million cases of hospital-transmitted infections occur in China every year, causing huge economic losses and treatment pressure, and primary hospital infections are seriously threatening the life and health safety of patients [1]. In addition, with the increase of irregular use and even abuse of antibacterial drugs, the occurrence of bacterial resistance is gradually increasing [2]. Therefore, the preparation of new antibacterial materials to overcome the limitations of traditional antibiotics prone to resistance and inhibit the spread of bacteria has become an important research direction in the field of biomedicine at present [3]. Among these novel antimicrobial materials, preparation of metal nanomaterials is a very effective process to develop antimicrobial agents for the inhibition of nosocomial bacteria. Sun et al. [4] developed an AuNR-MgHA scaffold and showed that the AuNR-MgHA scaffold was able to reduce bacteria in suspension and adhesion by 24% after 100 h and showed good antibacterial performance against both *S. aureus* and *E. coli*. Biao et al. [5] used chitosan to synthesize stable Ag colloids in aqueous solution, and antibacterial tests showed that the synthesized chitosan-Ag colloids at pH 5.0 had high bactericidal efficiency against both bacteria and fungi. Vinit Prakash et al. [6] describes the synthesis of copper nanoparticles from a hydroethanolic extract of *P. kurroa* rhizomes (CuNPs-Pk) and their evaluation for antimicrobial activities against *E. coli* and *S. aureus.* In addition, many nanoparticles have recently been developed for the treatment of bacterial infections. However, most of the reported nanomaterials have significant toxicity and also require multi-step synthesis processes [7]. Therefore, there is an increasing need for a simpler and more effective synthetic method and safe antimicrobial agents to inhibit bacteria.

*Staphylococcus aureus*, *Escherichia coli*, *Pseudomonas aeruginosa*, *Acinetobacter baumannii*, *Acinetobacter junii* and *Staphylococcus epidermidis* are all common strains of hospital-acquired infections. Of these, *S. aureus* is one of the most common pathogens, causing a wide range of disease symptoms and infections worldwide. Such infections can cause thousands of deaths if improperly treated with medications [8]. *E. coli* is a conditional pathogen that can cause prostate, gallbladder and bloodstream infections in nosocomial infections, as well as pneumonia and, in newborns, meningitis [9]. *P. aeruginosa*, also known as *Pseudomonas aeruginosa*, is a common clinical opportunistic pathogen causing serious nosocomial infections, mostly in immunocompromised states (e.g., trauma, burns, heavy use of antibiotics or immunosuppressive agents) causing a variety of acute and chronic infections [10]. *A. baumannii* and *A. junii*, are widely distributed in the hospital environment and are prone to colonize the skin, conjunctiva, oral cavity, respiratory tract, gastrointestinal tract and genitourinary tract of hospitalized patients, and infections can occur when the immunity of the body is reduced [11]. *S. epidermidis* is an important causative agent of medical device-related hospital-acquired infections, and its infection is difficult to treat because of its drug resistance rate of up to 90% [12]. To overcome nosocomial infections caused by multiple bacteria, there is an urgent need to develop a new antimicrobial agent.

More metals and their released ions have the effect of inhibiting and killing microorganisms, but since most metal ions are harmful to humans, the ones currently being used more as antimicrobial agents are silver, copper and zinc [13,14,15]. Zinc has good antibacterial effect, as early as in ancient Egypt, human in the form of zinc carbonate ore for local anti-inflammatory and antiseptic, and later on zinc oxide, zinc sulfate and zinc stearate powder or ointment for local use [16]. Zinc ion is an excellent antibacterial agent, but its use is somewhat limited because its antibacterial effect is inferior to that of silver and copper [17]. However, at the same time, zinc or zinc oxide has the advantages of small size effect of raw materials, surface effect, cheap price, abundant resources and non-toxic effect on the environment, etc. [18]. Currently, antimicrobial materials containing zinc or zinc oxide have been gradually applied in many fields such as food packaging, textiles, biomedicine, etc. [19,20,21]. For example, in the area of medical devices, Petkova et al. [22] have successfully synthesized ZnO thin films on medical textiles using a simultaneous sonochemical-enzymatic coating method. ZnO nanoparticle (NP)-coated fabrics have effectively inhibited the growth of *E. coli* and *S. aureus* by 67% and 100%, respectively. Shim, K. et al. [23] have prepared ZnO NP structures with different surface roughnesses, wettability and concentrations using an RF magnetron sputtering system. ZnO NP coatings have achieved 99.5% inhibitions for the growth of multiple bacteria, namely *E. coli*, *S. aureus* and *P. funiculosum*. Maimaiti et al. [24] produced coatings of nano-HA (nHA) and ZnO-NPs. This coating exhibited good antibacterial properties while retaining mineralization functionality.

Metal-Organic Frameworks (MOFs) are a new class of porous inorganic−organic materials through the composition between organic ligands and metal ions or clusters, which play an important role in water treatment [25], biosensors [26], electrode materials [27], catalysis [28], adsorption [29,30], diagnostics [31], promotion of wound healing [32] and drug delivery [33,34]. With the continuous development of nanomaterial technology, MOF-based antimicrobial materials have become a hot spot for antimicrobial applications [35]. Compared with traditional antimicrobial agents, they have the following advantages: (i) MOFs with different metal centers can gradually release silver, zinc, iron, copper and cobalt ions through the degradation process to achieve a lasting antimicrobial effect; (ii) the chelation of MOFs reduces the polarity of metal ions, which enhances their lipophilicity and facilitates the penetration of MOFs through bacterial cell membranes to sterilize MOFs; (iii) the high porosity and specific surface area can promote the effective wrapping/loading of other materials into their pores; (iv) the low toxicity and good biocompatibility of MOFs promote their application in various aspects [36,37,38]. There have been many recent studies on loading metal ions into MOFs. For example. Tian, M.et al. [39] have successfully constructed a hydrogel wound dressing consisting of the bimetallic MOF loaded with glucose oxidase (GOx), termed MOF(Fe-Cu)/GOx-polyacrylamide (PAM) gel. The catalytic activity of the bimetallic MOF(Fe-Cu) is significantly enhanced due to doping of copper, which makes it possess an outstanding antibacterial ability based on cascade catalysis. Verma, A et al. [40] have composed Ag NCs by using MOFs as scaffold. The nanocomposite was highly bactericidal against both Gram−positive and Gram−negative bacterial strains and caused cell wall perforation. Barjasteh, M. et al. [41] have synthesized a novel zeolitic imidazolate framework-8 (ZIF-8) functionalized with Ag (Ag@ZIF-8) NPs. The Ag-containing wound dressings showed 52–300% of effective antibacterial activities. Although the addition of metal ions can improve the sterilization ability of MOFs, the process has some drawbacks, such as cytotoxicity by high doses of metal ions, slow sterilization rate and non-persistent sterilization effect [42,43,44].

In this study, we designed a ZnO-doped derivative of MIL-101(Fe) with efficient ion release, good antibacterial properties, good biosafety and which can be used for broad-spectrum sterilization. We characterized the structure of MIL-101(Fe)@ZnO by FTIR, ultraviolet–visible(UV-vis), ICP-OES, XRD, scanning electron microscopy(SEM), energy dispersive X-ray spectrometry(EDS) and Brunauer-Emmett-Teller(BET) analysis. The inhibition performance of MIL-101(Fe)@ZnO against *S. aureus*, *E. coli*, *P. aeruginosa*, *A. baumannii*, *A. junii* and *S. epidermidis* was investigated by bacterial survival experiments and bacterial growth curves. The antibacterial mechanism was also explored using reactive oxygen species (ROS) generation, protein leakage and lipid preoxidation, and finally, the biosafety of the nanomaterials was explored at the cellular level, showing that the synthesized materials can be used as attractive and promising antibacterial agents for the inhibition of a wide range of bacteria. To the best of our knowledge, this is the first report on MOFs incorporating MIL-101(Fe) and ZnO as effective antimicrobial agents against a wide range of bacteria.

## 2. Results

### 2.1. Characterization

**Figure 1:**
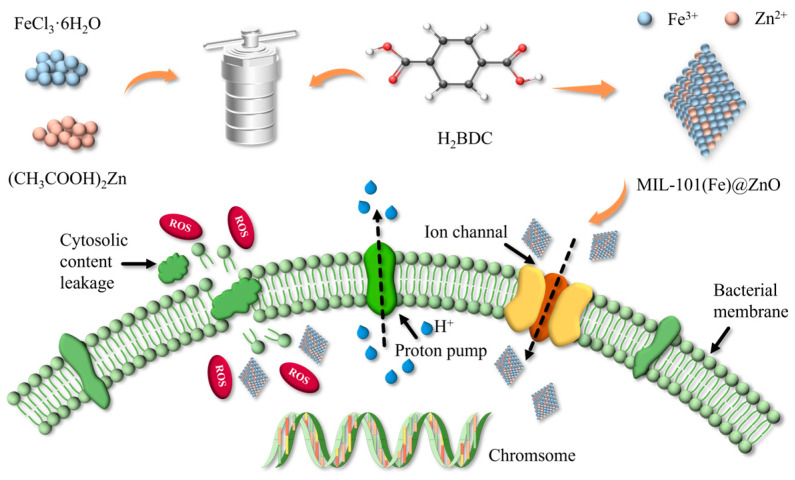
Schematic illustration of the whole reaction process and possible antibacterial mechanism of MIL-101(Fe)@ZnO.

MIL-101(Fe)@ZnO was synthesized by the solvothermal synthesis method. First, H_2_BDC and FeCl_3_·6H_2_O were reacted in DMF, and then (CH_3_COO)_2_Zn was added. The nanocomposites were obtained under high temperature and high pressure conditions in the reactor. The schematic diagram of the whole reaction process is shown in Figure 1. The synthesized MIL-101(Fe)@ZnO nanoparticles were characterized and analyzed by FT-IR, UV-vis, ICP-OES, XRD, SEM, EDS and BET.

#### 2.1.1. FT-IR Spectroscopy

**Figure 2:**
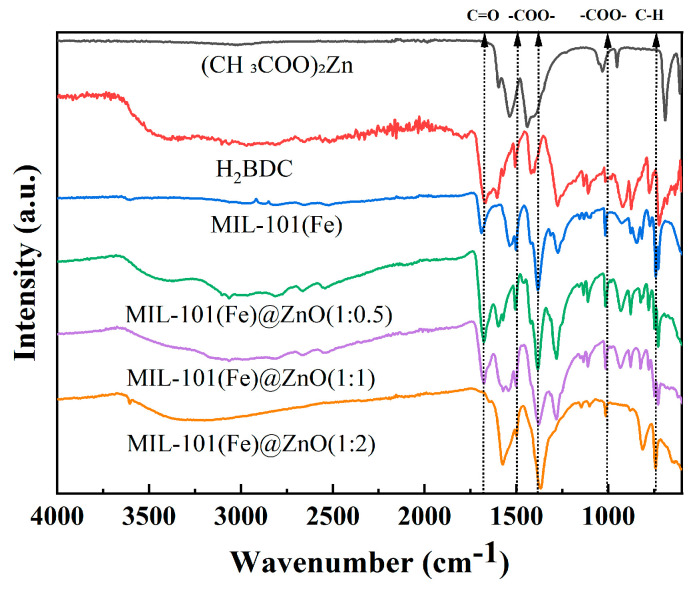
FT-IR spectra of (CH_3_COO)_2_Zn, H_2_BDC, MIL-101(Fe) and MIL-101(Fe)@ZnO.

To verify ZnO modification on MIL-101(Fe), FT-IR spectrograms of (CH_3_COO)_2_Zn, H_2_BDC, MIL-101(Fe) and MIL-101(Fe)@ZnO were recorded [45]. As shown in Figure 2, the peaks at 1438 cm^−1^ and 1539 cm^−1^ in the IR spectrum of (CH_3_COO)_2_Zn correspond to the symmetric and antisymmetric stretching vibrations of C=O, respectively. In the IR spectrum of H_2_BDC, the peak at 723 cm^−1^ is attributed to the C-H bond in benzene, the peaks at 1419 cm^−1^ and 1508 cm^−1^ correspond to the carboxyl bond and the peak at 1672 cm^−1^ originates from the stretching vibration of C=O in the carboxyl functional group. The IR spectrum of MIL-101(Fe) has characteristic peaks of (CH_3_COO)_2_Zn and H_2_BDC, where the peak at 743 cm^−1^ is attributed to the C-H bond in benzene, the peaks at 1017 cm^−1^, 1386 cm^−1^ and 1504 cm^−1^ correspond to the carboxyl bond and the peak at 1691 cm^−1^ originates from the stretching vibration of C=O in the carboxyl functional group. Compared with the IR spectrum of MIL101(Fe), the characteristic absorption peaks of MIL-101(Fe)@ZnO appear at 731 cm^−1^, 1017 cm^−1^, 1386 cm^−1^, 1504 and 1681 cm^−1^, indicating that the presence of MIL-101(Fe)@ZnO loaded with Zn ions still exists on MIL-101(Fe), while the intensity of the absorption peak at 1681 cm^−1^ is enhanced and the absorption peak of the stretching vibration of -OH at wave numbers between 3651 and 3300 cm^−1^ is also significantly enhanced, and these changes prove that Zn may be bound to MIL-101(Fe) in the form of intermolecular hydrogen bonding. Figure 2 also shows that the peak sites gradually disappear as the amount of added zinc ions increases, and even a shift of the peak sites occurs after a large dose of added zinc. Accordingly, we believe that the disappearance of some characteristic peaks is mainly due to the combination of the added zinc ions with oxygen forming zinc oxide, and the oxygen ions in the zinc oxide will affect the oxygen-containing functional groups in MIL-101(Fe), resulting in the limitation of the synthesized product MIL-101(Fe). In order to add the target metal without affecting the synthesis of MIL-101(Fe), we believe that we need to select a suitable ratio of MIL-101(Fe) to zinc.

#### 2.1.2. UV-Vis Spectroscopy

**Figure 3:**
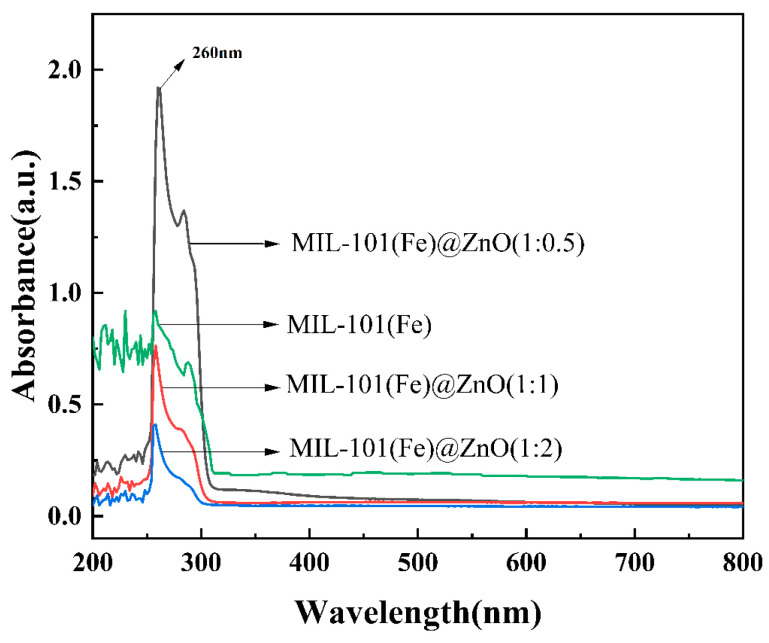
UV spectra of MIL-101(Fe) and MIL-101(Fe)@ZnO.

UV-vis absorption spectra of MIL-101(Fe), MIL-101(Fe)@ZnO were detected using dimethyl sulfoxide (DMSO) as a reference [46]. As shown in Figure 3, the characteristic absorption peaks of three different mass ratios of MIL-101(Fe)@ZnO were all at 260 nm, which was consistent with that of MIL-101(Fe), further indicating the presence of MIL-101(Fe) in MIL-101(Fe)@ZnO. In addition, Figure 3 also shows that MIL-101(Fe) exhibits an obvious UV peak in the range of 200–300 nm, and the structure of MIL-101(Fe) begins to collapse as the amount of added zinc ions increases, with the gradual disappearance of the outgoing peak site. This also indicates that the amount of added zinc acetate to MIL-101(Fe) is limited, and is in agreement with the above results of the FTIR.

#### 2.1.3. ICP-OES

**Figure 4:**
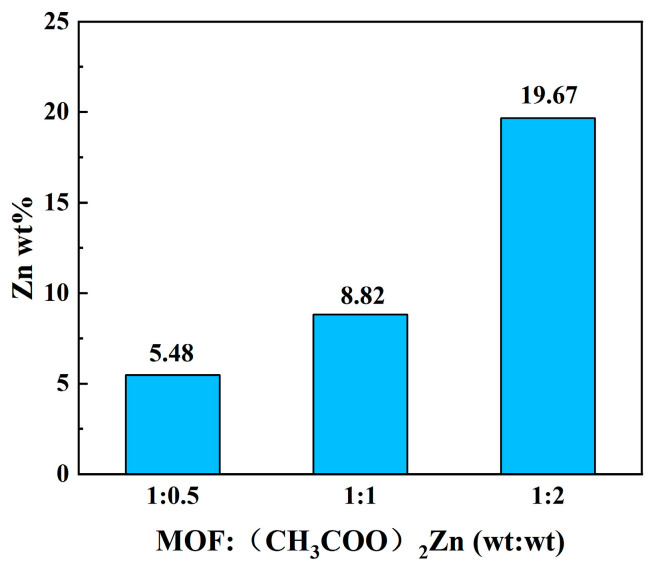
Zine loading of MIL-101(Fe)@ ZnO with different mass ratios.

As shown in Figure 4, the actual content of Zn in the MIL-101(Fe)@ZnO synthesized with different mass ratios was determined, where the contents of Zn in MIL-101(Fe)@ZnO with mass ratios of 1:0.5, 1:1 and 1:2 were 5.48%, 8.82% and 19.67%, respectively [46].

#### 2.1.4. XRD

**Figure 5:**
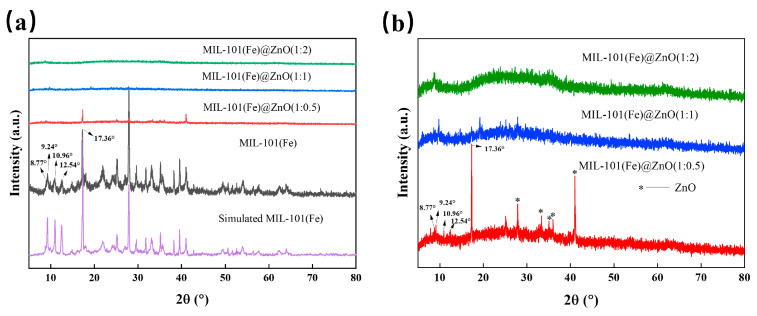
(**a**) Comparison of the XRD spectra of MIL-101(Fe), simulated MIL-101(Fe) and MIL-101(Fe)@ZnO. (**b**) Comparison of the XRD spectra of MIL-101(Fe)@ZnO.

The X-ray diffractograms of MIL-101(Fe), MIL-101(Fe)@ZnO are recorded in Figure 5 [47]. As shown in Figure 5a, the intensity of each diffraction peak of MIL-101(Fe) is high and the spectral peaks are sharp, showing high crystallinity. The five main diffraction peaks with 2θ values of 8.77°, 9.24°, 10.96°, 12.54° and 17.36°, are matched well with the simulated pattern and the other previous report, indicating that the synthesized material is pure MIL-101(Fe) [48,49].

Figure 5b shows the XRD patterns of three different weight ratios of MIL-101(Fe) @Zn. With the increase of Zn acetate addition, the position of the MIL-101(Fe) diffraction peaks remained the same, but their relative intensity gradually decreased. The lower crystallinity of the XRD patterns of MIL-101(Fe)@ZnO(1:1) and MIL-101(Fe)@ZnO(1:2) may be attributed to the fact that the addition of zinc acetate in excess affects the crystallization process of MIL-101(Fe), which causes the structure of MIL-101(Fe) to collapse. Moreover, the peaks were too low to determine whether ZnO was incorporated or not, in agreement with the above FTIR and UV-vis results. However, the XRD pattern of MIL-101(Fe)@ZnO(1:0.5) still had more obvious MIL-101(Fe) diffraction peaks. Meanwhile, 2θ = 27.87°, 33.33°, 35.07°, 36.01°, 41.01° on MIL-101(Fe)@ZnO(1:0.5) correspond to the diffraction peaks of the ZnO standard card (JCPDS 36-1451) [48]. The results indicate that Zn can be doped into the MIL-101(Fe) lattice to form a bimetal and has little effect on the skeleton topology of MIL-101(Fe) when the weight ratio is 1:0.5. Therefore, in combination with the above characterization results, MIL-101(Fe)@ZnO(1:0.5) with distinctive diffraction peaks and high crystallinity will be selected for subsequent experimental studies.

#### 2.1.5. SEM and EDS Analysis

**Figure 6:**
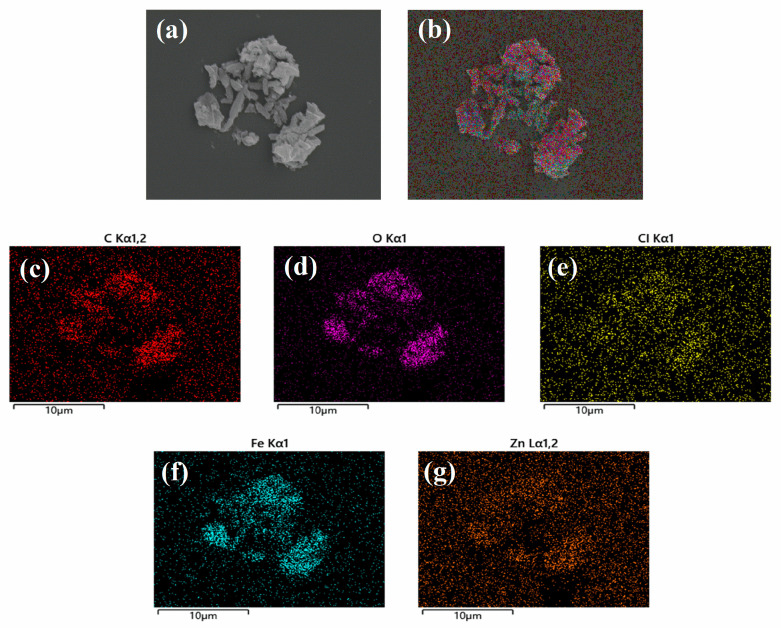
(**a**) SEM images of MIL-101(Fe)@ZnO(1:0.5). (**b**–**g**) EDS for the elemental color mappings (C, O, Cl, Fe, Zn) spectrum of MIL-101(Fe)@ZnO(1:0.5).

SEM was used to observe the morphology of MIL-101(Fe)@ZnO(1:0.5) [50]. As shown in Figure 6a, MIL-101(Fe)@ZnO(1:0.5) has a rectangular structure with a heterogeneous particle size and easy agglomeration. Figure 6b–g shows the elemental analysis of MIL-101(Fe)@ZnO(1:0.5), from which it can be seen that five elements, Zn, Fe, C, Cl and O, are uniformly distributed in the NPs. Among these elements, C, O, Cl and Fe illustrate the establishment of the MIL-101(Fe) skeleton centered on trivalent iron, while Zn is the characteristic element of MIL-101(Fe)@ZnO(1:0.5). Meanwhile, the accurate quantitative analysis of the Fe and Zn elements of MIL-101(Fe)@ZnO(1:0.5) was also performed using the ICP-OES method. The results showed that the contents of Fe and Zn elements were 30.82% and 5.48%, respectively, so this result further proved the successful synthesis of MIL-101(Fe)@ZnO(1:0.5).

#### 2.1.6. BET Analysis

**Figure 7:**
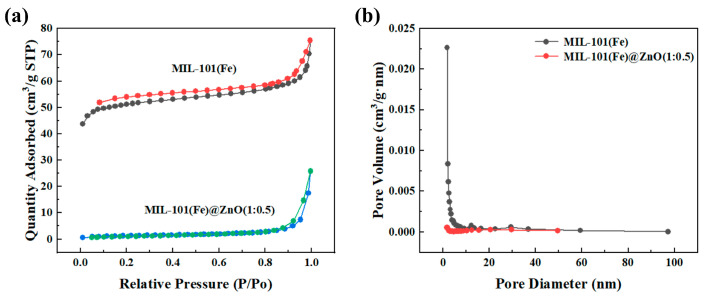
(**a**) N_2_ adsorption−desorption isotherms of MIL-101(Fe) and MIL-101(Fe)@ZnO(1:0.5). (**b**) BJH pore size distribution curve of MIL-101(Fe) and MIL-101(Fe)@ZnO(1:0.5).

The BET surface area and pore structure were evaluated by N_2_ adsorption−desorption experiments. As can be observed in Figure 7a, MIL101(Fe) belongs to the type IV N_2_ adsorption isotherm, indicating that MIL-101(Fe) has a porous structure [48]. Combined with Figure 7b and the specific surface area and pore size analysis, this shows that the specific surface area of MIL101(Fe) is 199.7 m^2^/g, the average pore size is 5.6 nm and the pore volume is 0.1 cm^3^/g. After the addition of ZnO, the specific surface area of MIL101(Fe)@Zn(1:0.5) is 4.7 m^2^/g, the average pore size is 20.5 nm, and the pore volume is 0.04 cm^3^/g. Among them, the specific surface area and pore volume decreased and the pore size increased because the ZnO NPs grown on the surface of MIL101(Fe) blocked the metal skeleton pore size, but this dispersed distribution could increase the contact area between them, which in turn enhanced the antibacterial activity of MIL101(Fe)@Zn(1:0.5).

### 2.2. Effect of MIL-101(Fe)@ZnO(1:0.5) on Bacterial Growth

#### 2.2.1. Metabolic Activity

**Figure 8:**
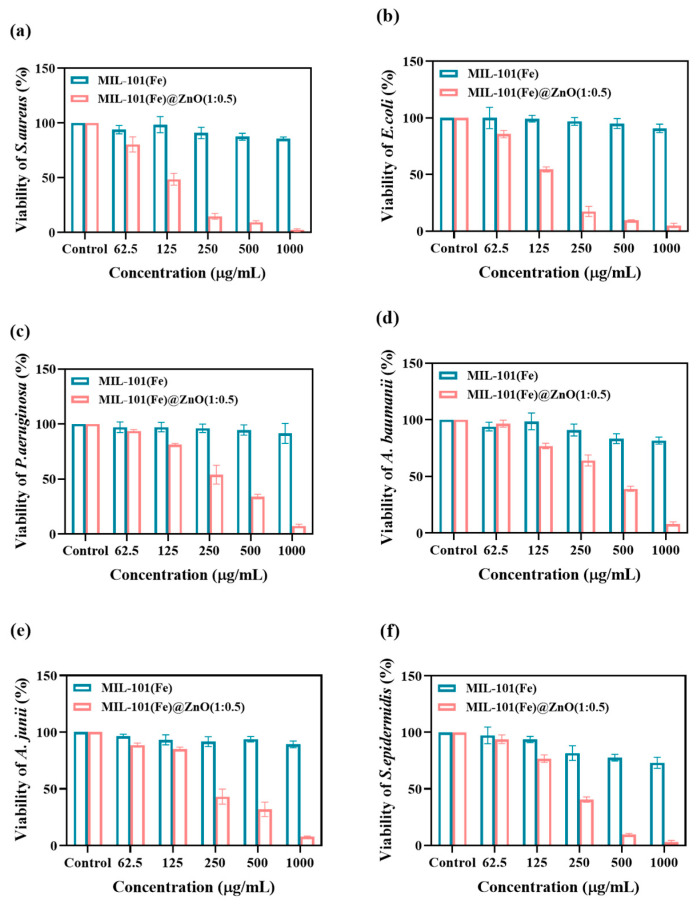
Results of metabolic activity with addition of MIL-101(Fe)@ZnO(1:0.5) and MIL-101(Fe) for inhibition of (**a**) *S. aureus.* (**b**) *E. coli.* (**c**) *P. aeruginosa.* (**d**) *A. baumannii.* (**e**) *A. junii.* and (**f**) *S.epidermidis*.

The MTT colorimetric analysis, pioneered by Mosmann [51], is simple, rapid, sensitive and stable, and is widely used in cell biology research. As shown in Figure 8, the concentration of MIL-101(Fe)@ZnO(1:0.5) showed a dose−response relationship with the survival of six pathogenic bacteria, as the concentration of MIL-101(Fe)@ZnO(1:0.5) increased, its ability to inhibit bacterial growth increased. The inhibition rate of MIL-101(Fe)@ZnO(1:0.5) at 250 μg/mL for *S. aureus, E. coli, P. aeruginosa, A. baumannii*, *A. junii* and *S. epidermidis* was 85.16%, 82.54%, 45.98%, 35.93%, 56.70% and 59.50%, respectively. At 500 μg/mL, the inhibition rates were 90.60%, 90.57%, 65.88%, 60.96%, 68.06% and 90.24%, respectively, with a more obvious antibacterial effect. Moreover, at 1000μg/mL, the inhibition rates of all six pathogenic bacteria were greater than 90%, which were 97.86%, 94.97%, 92.71%, 91.91%, 92.18% and 97.09%, respectively. Meanwhile, the bacterial inhibition ability of MIL-101(Fe)@ZnO(1:0.5) combined with zinc ions was found to be substantially higher compared with that of pure MIL-101(Fe). As shown by the results of MTT experiments, MIL-101(Fe)@ZnO(1:0.5) showed good antibacterial effects against all six pathogenic bacteria and the activity against Gram-positive (G+) bacteria was higher than that of Gram-negative (G-) bacteria.

#### 2.2.2. Bacterial Growth Curve

Bacterial growth curves reflect the population growth pattern of single-celled microorganisms when they are cultured in liquid under certain environmental conditions. Depending on their growth rates, the growth curves can be generally classified into delayed, logarithmic, stable and decaying phases [52,53].

**Figure 9:**
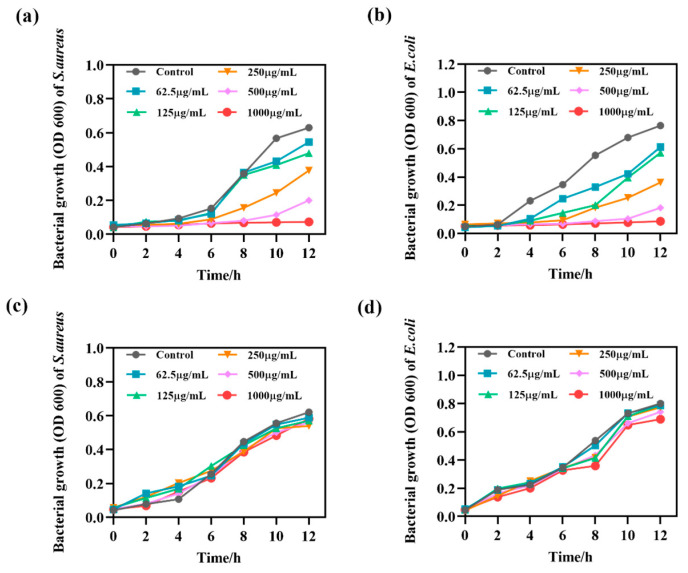
Bacterial growth curves of MIL-101(Fe) and MIL-101(Fe)@ZnO(1:0.5) for inhibition of *E. coli* and *S. aureus* growth. (**a**) Bacterial growth curve of inhibition of *S. aureus* growth by MIL-101(Fe)@ZnO(1:0.5). (**b**) Bacterial growth curve of inhibition of *E. coli* growth by MIL-101(Fe)@ZnO(1:0.5). (**c**) Bacterial growth curve of inhibition of *S. aureus* growth by MIL-101(Fe). (**d**) Bacterial growth curve of inhibition of *E. coli* growth by MIL-101(Fe).

As shown in Figure 9, the growth curve of bacteria treated with MIL-101(Fe) was similar to that of the normal control, while bacteria treated with MIL-101(Fe)@ZnO(1:0.5) entered the logarithmic growth phase later and eventually entered the decay phase with lower OD600 values. At MIL-101(Fe)@ZnO(1:0.5) of 1000 μg/mL entered the logarithmic growth phase after 10 h. The normal control group of *S. aureus* and *E. coli* entered the logarithmic growth phase after 6 h and 2 h, respectively, MIL-101(Fe)@ZnO(1:0.5) delayed the entry of *S. aureus* and *E. coli* into the logarithmic growth phase by 4 h and 6 h, while the bacterial growth trend was flatter and the OD value in the decay phase was lower. The results indicated that the growth of bacteria in MIL-101(Fe)@ZnO(1:0.5) was significantly inhibited and showed a dose−response relationship. The results of the growth curves were consistent with the MTT, once again indicating that the synthesized MIL-101(Fe)@ZnO(1:0.5) has good bacterial inhibitory activity.

### 2.3. Investigation of the Antibacterial Mechanism

To explore the antibacterial mechanism of MIL-101(Fe)@ZnO(1:0.5), we examined the bacterial morphology using SEM as well as measured the levels of ROS, protein leakage and lipid peroxidation, and the possible antimicrobial mechanisms are shown in Figure 1.

#### 2.3.1. Bio-SEM

**Figure 10:**
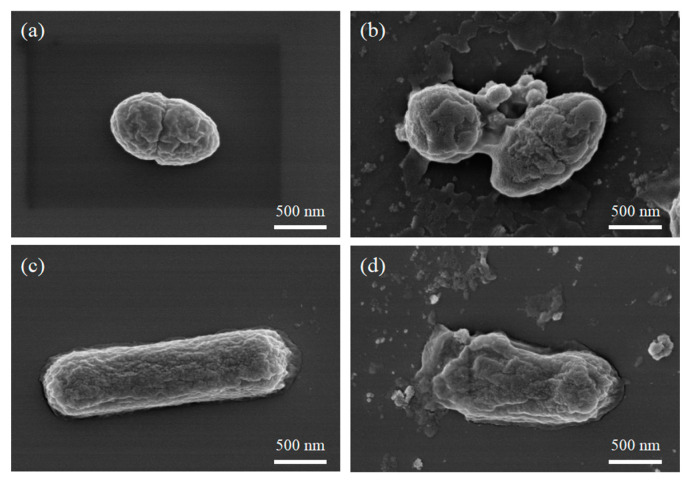
SEM image of normal *S. aureus* (**a**) and *E. coli* (**b**); *S. aureus* (**c**) and *E. coli* (**d**) induced by treatment with MIL-101(Fe)@ZnO(1:0.5).

Effect of MIL-101(Fe)@ZnO(1:0.5) on the structure and morphology of bacterial biofilm by SEM [54]. As shown in Figure 10, all normal bacterial cells showed an intact and smooth cellular state, while both *S. aureus* and *E. coli* cells treated with MIL-101(Fe)@ZnO(1:0.5) showed a dry and damaged state, with severe cellular damage and leakage of intracellular material observed. The SEM results again demonstrated that MIL-101(Fe)@ZnO(1: 0.5) can strongly interact with pathogenic bacteria and kill them through cellular damage [55].

#### 2.3.2. Determination of the ROS

**Figure 11:**
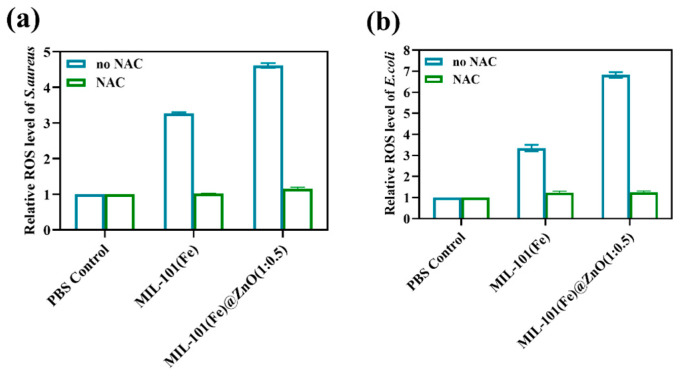
Intracellular ROS production by *S. aureus* (**a**) and *E. coli* (**b**) induced by treatment with MIL-101(Fe) and MIL-101(Fe)@ZnO(1:0.5). The content of ROS in all of the treatment groups was normalized to 1 with the control group.

Oxygen is essential for aerobic organisms to respire or obtain energy from nutrients, and is also a precursor for ROS such as superoxide anion radicals (O^2−^), hydrogen peroxide (H_2_O_2_) and highly reactive hydroxyl radicals (O). These highly reactive substances react with biologically important molecules such as lipids, proteins and nucleic acids, ultimately leading to oxidative damage and even death of the organism [56,57,58]. As shown in Figure 11, MIL-101(Fe)@ZnO(1:0.5)-treated bacteria induced more ROS production than MIL-101(Fe)-treated bacteria, and when the ROS inhibitor NAC was added, the enhanced effect induced by MIL-101(Fe)@ZnO(1:0.5) and MIL-101(Fe) disappeared and the intracellular ROS content in bacteria returned. The results indicated that MIL-101(Fe)@ZnO(1:0.5) induced the production of intracellular ROS, leading to the destruction of cell structure and eventually to bacterial death.

#### 2.3.3. Bacterial Protein Leakage

**Figure 12:**
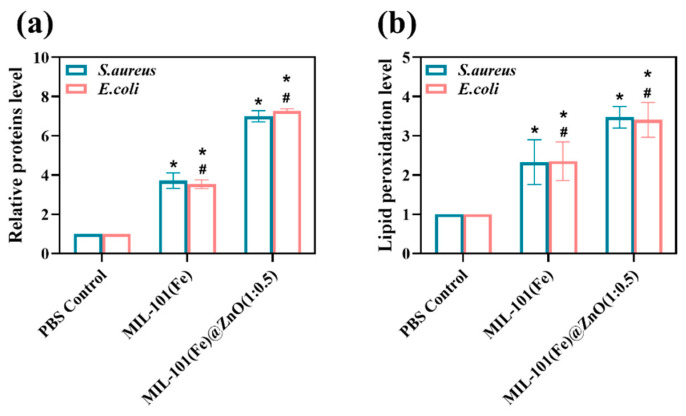
Relative proteins level (**a**) and lipid peroxidation level (**b**) by *S. aureus* and *E. coli* induced by treatment with MIL-101(Fe) and MIL-101(Fe)@ZnO(1:0.5). The content of relative proteins level or lipid peroxidation levels in all of the treatment groups was normalized to 1 with the control group. * means (*p* < 0.05), # means (*p* < 0.05).

MIL-101(Fe)@ZnO(1:0.5) significantly accelerated the membrane leakage and facilitated the subsequent protein leakage [59]. Figure 12a revealed that MIL-101(Fe)@ZnO(1:0.5) enhances the membrane leakage of proteins. No protein was detected in the blank control group, and the difference between the MIL-101(Fe) group and the blank control group was not significant. Protein leakage from bacterial cells co-cultured with MIL-101(Fe)@ZnO(1:0.5) was significantly increased compared to the control and MIL-101(Fe) groups (*p* < 0.05).

#### 2.3.4. Lipid Peroxidation Levels

The production of ROS subsequently causes lipid peroxidation damage to the cell membrane and reduces membrane fluidity [59]. As shown in the Figure 12b, MIL-101(Fe)@ZnO(1:0.5) showed higher levels of lipid peroxidation compared to MIL-101(Fe), indicating that MIL-101(Fe)@ZnO(1:0.5) treatment generated more ROS, which is consistent with intracellular ROS measurements (Figure 11).

### 2.4. Cytotoxicity Test

**Figure 13:**
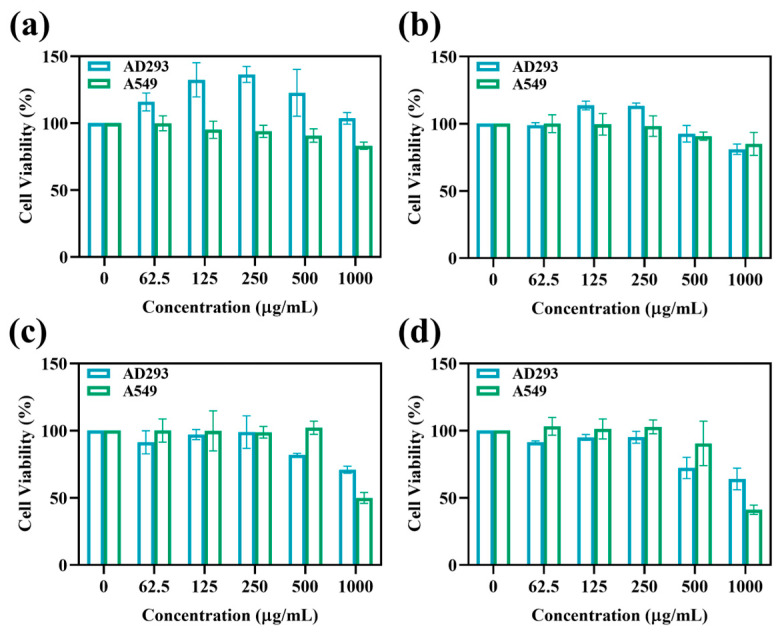
Survival rate of MIL-101(Fe)@ZnO(1:0.5) -treated AD293 and A549 cells after 12 h (**a**), 24 h (**b**), 48 h (**c**) and 72 h (**d**).

The safety evaluation study of MIL-101(Fe)@ZnO(1:0.5) provides an important reference value for its wide application in related fields [43,60]. The cytotoxicity of MIL-101(Fe)@ZnO(1:0.5) was determined by MTT assay using AD293 and A549 cells. As shown in Figure 13, the cell viability of MIL-101(Fe)@ZnO(1:0.5) was maintained above 100% after 12 h of co-culture with AD293 cells in a range of concentrations examined experimentally, and after 24 h, 48 h and 72 h of co-culture, the cell viability was, respectively, 90%, 80% and 70% even at high concentrations of 500 μg/mL. Meanwhile, the cell survival rate of MIL-101(Fe)@ZnO(1:0.5) remained above 90% after co-culture with A549 cells for 12 h, 24 h, 48 h and 72 h at a high concentration of 500 μg/mL, demonstrating the good biocompatibility of MIL-101(Fe)@ZnO(1:0.5).

### 2.5. Hemolytic Assay

**Figure 14:**
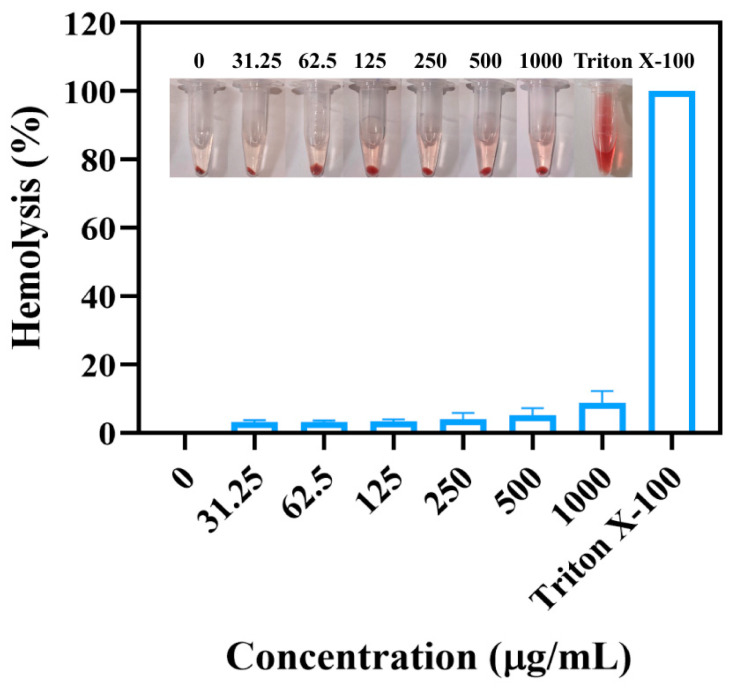
Hemolysis analysis of MIL-101(Fe)@ZnO(1:0.5). Physiological saline and 0.1% Triton X-100 were used as negative and positive controls, respectively.

The ability of MIL-101(Fe)@ZnO(1:0.5) to lyse erythrocytes can be used as a basic characterization of biocompatibility, and if it can damage erythrocytes, the erythrocytes will release heme, thus making the system red [61]. As shown in Figure 14, it is obvious from the pictures taken that in the positive control 1% TritonX-100, erythrocytes are completely destroyed and the supernatant appears red, while in each concentration of MIL-101(Fe)@ZnO(1:0.5) and PBS negative control, erythrocytes are intact and the supernatant is colorless and transparent, and the hemolysis rate of erythrocytes is below 10%, and the hemolysis rate is maintained at a low. The hemolysis rate was maintained at a low level, so it has good hemocompatibility. Combined with the above cytotoxicity experiments, it can be proved that MIL-101(Fe)@ZnO(1:0.5) has good biocompatibility.

## 3. Discussion

In this study, we synthesized the target product MIL-101(Fe)@ZnO by solvothermal synthesis. We evaluated the antibacterial activity, antibacterial mechanism and biosafety of MIL-101(Fe)@ZnO(1:0.5). FTIR, UV-vis, ICP-OES, XRD, SEM, EDS and BET showed that ZnO was successfully encapsulated in MIL-101(Fe) and MIL-101(Fe)@ZnO(1:0.5) had distinct characteristic diffraction peaks and high crystallinity, without structural collapse [18,62,63].

The antimicrobial experiments showed that MIL-101(Fe)@ZnO(1:0.5) showed good antimicrobial effects against all six pathogenic bacteria compared to MIL-101(Fe). Interestingly, MIL-101(Fe)@ZnO(1:0.5) showed higher activity against G+ bacteria than G-, which may be related to differences in its cell wall composition. In contrast to G+ bacteria, G- bacteria have an additional lipopolysaccharide (LPS) layer in the cell wall composition. LPS has some selective uptake function that allows the permeation of small molecules such as pyrimidines, purines, peptides, amino acids, etc., and blocks the entry into the cell of macromolecules such as lysozyme, penicillin and decontaminants [64]. From this, it can be hypothesized that G- bacteria can also block the entry of MIL-101(Fe)@ZnO(1:0.5) into the cell to a certain extent, resulting in its weaker activity against G- bacteria.

Antimicrobial mechanism experimentally demonstrated that the antimicrobial mechanism (Figure 1) of MIL-101(Fe)@ZnO(1:0.5) includs: (i) MIL-101(Fe)@ZnO(1:0.5) acts as a reservoir of Zn^2+^ to provide a slow release of Zn^2+^, and the Zn^2+^/Fe^3+^ is released and attached to the negatively charged cell membranes, which causes mechanical damage to the microbial cell walls through electrostatic interactions, so MIL-101(Fe)@ZnO(1:0.5) has a long-lasting antimicrobial effect. (ii) The production of ROS acts as a free radical compound and a non-selective oxidant, destroying most biomolecules, including lipids and amino acids, both of which make up the bacterial cell wall. (iii) Leakage of cell contents due to membrane disruption can lead to cell membrane contraction and cell lysis [57,59]. In addition to this, the results of the antibacterial mechanism showed that the levels of ROS production, protein leakage and lipid preoxidation were higher for MIL-101(Fe)@ZnO(1:0.5) than for MIL-101(Fe). This phenomenon illustrates the existence of synergistic effects between bimetallic ions leading to stronger bactericidal effects compared to single metals. Meanwhile, bimetallic materials usually have higher catalytic activity and selectivity than monometallic materials [16,37,44].

Zn^2+^ is an endogenous low-toxicity transition metal cation [65]. In the cytotoxicity assay, the survival rate of AD293 cells and A549 cells decreased gradually with the gradual increase of MIL-101(Fe)@ZnO(1:0.5) concentration, but the cell viability remained at a high level. The cell survival rates remained above 70% and 90%, respectively, indicating that MIL-101(Fe)@ZnO(1:0.5) was non-toxic to AD293 and A549 cells. Hemolysis experiments also showed that the hemolysis rate of MIL-101(Fe)@ZnO(1:0.5) on erythrocytes was maintained at a low level, thus indicating that MIL-101(Fe)@ZnO(1:0.5) has good biocompatibility.

However, since the bactericidal effect of zinc is moderate compared to other metals such as silver and copper, it needs to be at a higher concentration to achieve efficient bacterial inhibition [17,21]. For example, MIL-101(Fe)@Ag(100 μg/mL) [66] has a lower MIC value than MIL-101(Fe)@ZnO (500 μg/mL) against *S. aureus* because it is encapsulated with silver ions that have superior antibacterial properties, but MIL-101(Fe)@Ag has a higher cytotoxicity. In addition, MIL-101(Fe)@ZnO(1:0.5) has a comparable or better antibacterial effect when compared with some MOF also synthesized on the basis of Zn^2+^. For example, the Zn-MOF synthesized by Akbarzadeh, F et al. [67] showed good inhibition of *A. baumannii* and *E. coli* at 500 μg/mL. In contrast, BioMIL-5 synthesized by Tamames-Tabar, C et al. [65] required 1.7 mg/mL to effectively inhibit *S. aureus* and *S. epidermidis.* Apart from that, Ni-MOF synthesized by Raju, P et al. [68] based on Ni^2+^ also required 1 mg/mL for effective inhibition of *P. aeruginosa*. 

After comparison, it can be concluded that the MIL-101(Fe)@ZnO(1:0.5) synthesized in this study has superior antimicrobial properties and is also a safe and green nanomaterial. The antimicrobial effect of MIL-101(Fe)@ZnO(1:0.5) can be further explored to develop a commercial antimicrobial agent with non-toxic and high bactericidal efficiency.

## 4. Materials and Methods

### 4.1. Materials

AD293 and A549 cells were purchased from the American Type Culture Collection (Manassas, VA, USA). Dulbecco’s modified Eagle medium, phosphate-buffered pH 7.4 (1×) 0.25% trypsin EDTA (1×) and penicillin–streptomycin (100,000 U/mL) were purchased from Gibco (Carlsbad, CA, USA). Fetal bovine serum was obtained from Shanghai ExCell Biologicals (Shanghai, China). Terephthalic acid (H_2_BDC, 99%), ferric chloride hexahydrate (FeCl_3_·6H_2_O, 99%), N,N-dimethylformamide (DMF, 99%), Glutaraldehyde solution (50%) and chicken erythrocytes (1%) were purchased from GBCBIO Technologies Inc. (Guangzhou, China). (CH_3_COO)_2_Zn (99%) and Glycerol (99%) were purchased from Aladdin Reagents Ltd. (Shanghai, China). The culture flasks were obtained from Corning Life Sciences Ltd. (New York, NY, USA). The 96-well plates were purchased from Guangzhou JITE Biofiltration Co. (Guangzhou, China). Luria–Bertani (LB) broth and nutritional agar medium were purchased from Guangdong Huan Kai Microbial Technology Co. (Guangzhou, China). The hydrothermal synthesis reactor was purchased from Zhengzhou Boke Instrument & Equipment Co. (Zhengzhou, China). The electrothermal blast thermostat oven (101-0B) was purchased from Shaoxing Licheng Instrument Technology Co. (Shaoxing, China). The Thermo-SorvallST16R frozen centrifuge was purchased from Thermo Fisher Scientific (Waltham, MA, USA).

### 4.2. Synthesis of MIL-101(Fe) and MIL-101(Fe)@ZnO

MIL-101(Fe) and MIL-101(Fe)@ZnO were synthesized according to previous reports [62]. A total of 2.702 g of FeCl_3_∙6H_2_O (10 mmol) and 0.830 g (5 mmol) of terephthalic acid (H_2_BDC) were dissolved in 60 mL of N,N-dimethylamide (DMF) solution and sonicated for 20 min. It was transferred to a 100 mL autoclave containing PTFE liner and heated continuously at 120 ℃ for 24 h to obtain a brick-red suspension, which was separated by centrifugation at 10,000 rmp for 10 min to obtain a brick-red precipitate. It was washed once with DMF and twice with hot anhydrous ethanol at 60 °C, and centrifuged at 10,000 rmp for 15 min to remove unreacted raw materials and DMF, respectively. Finally, the washed brick-red precipitate was dried in an electric blast oven at 120 °C for 12 h to obtain MIL -101(Fe).

Furthermore, in order to introduce ZnO into the MIL-101(Fe) framework, (CH_3_COO)_2_Zn was used as a source of ZnO. Moreover, the mass ratios of FeCl_3_∙6H_2_O to (CH_3_COO)_2_Zn were 1:0.5, 1:1 and 1:2. That means 2.702 g of FeCl_3_∙6H_2_O and 1.351 g of (CH_3_COO)_2_Zn were dissolved in 60 mL of DMF, and the final mass ratio of FeCl_3_∙6H_2_O to (CH_3_COO)_2_Zn was 1:0.5. The following steps were the same as those for the synthesis of MIL-101(Fe). 

### 4.3. Nanomaterial Characterization

Powder XRD (Rigaku Ultima IV, Kyoto, Japan) was performed using Cu-Kα radiation in the 2θ range from 5° to 80°. For FTIR spectroscopy (Bruker ALPHA II, Karlsruhe, Germany), the spectra of MIL-101(Fe), MIL-101(Fe)@ZnO, and (CH_3_COO)_2_Zn were recorded in the wavelength range of 600–4000 cm^−1^. An inductively coupled plasma (ICP) spectrometry generator was used to determine the Zn content in MIL-101(Fe)@ZnO. The UV–vis absorption spectra were measured with a HITACHI U-3010 UV–vis diffuse reflectance spectrophotometer (Hitachi Limited, Kyoto, Japan) using DMSO as a reference. The morphology, shape and size of MIL-101(Fe) and MIL-101(Fe)@ZnO were investigated with SEM (TESCAN Mira4, Brno, Czech Republic) at an accelerating voltage of 200 eV to 30 keV. The chemical composition of MIL-101(Fe)@ZnO was further analyzed with EDS (Zeiss EVO, Oberkochen, Germany). The BET surface area and porous structure were evaluated with N_2_ (77.4 K) adsorption-desorption experiments (Micromeritics ASAP 2020, Norcross, GA, USA).

### 4.4. Antimicrobial Activity

#### 4.4.1. Bacterial Culture

*Staphylococcus aureus* (*S. aureus*, ATCC 25923) and *Escherichia coli* (*E. coli*, ATCC 43894), *Pseudomonas aeruginosa* (*P. aeruginosa*, ATCC 27853), *Acinetobacter baumannii* (*A. baumannii*, ATCC 19606), *Acinetobacter junii* (*A. junii*, ATCC 17908) and *Staphylococcus epidermidis* (*S. epidermidis*, ATCC 12228) were purchased from ATCC. The bacteria were then inoculated onto nutrient agar plates by zonation and incubated for 24 h at 37 °C. Single colonies were picked from the plates with the inoculation loop in PBS, mixed well and turbidified by standard turbidimetric method to obtain 1 × 10^8^ CFU/mL bacterial suspension, which was diluted 100 times to obtain 1 × 10^6^ CFU/mL bacterial suspension.

#### 4.4.2. Bacterial Cell-Viability Assay

The viability of bacterial cells was measured by the 3-(4, 5-dimethylthiazol-2-yl) -2, 5-diphenyltetrazolium bromide (MTT) technique according to standard protocol [51]. In a 96-well plate, 150 μL samples at different concentrations were added, followed by the addition of equal amounts of diluted bacterial solution (1 × 10^6^ CFU/mL) per well in each well, and a control row with only sample and PBS, a negative row with only LB medium and equal amounts of PBS and a positive control with LB medium and equal amounts of bacterial solution only. The plates were incubated in a shaker (120 r/min, 37 °C) for 12 h. After that, 5 mg/mL of MTT solution was added to each well for 10 μL and incubated at 37 °C for 4 h. Then, 100 μL of DMSO solution was added to each well and shaken for 10 min to dissolve the methanogenic crystals. Finally, the absorbance value of 492 nm was measured by enzyme standardization instrument, and 570 nm was measured for reference. The antibacterial effect of MIL-101(Fe)@ZnO(1:0.5) was evaluated with the following equations:

Survival rate (%) = (OD test samples − OD negative control)/(OD positive control − OD negative control) × 100%(1)

Mortality rate (%) = 1 − Survival rate (%)(2)

where OD is the optical density value.

#### 4.4.3. Bacterial Growth Curve

In a 96-well plate, 150 μL of different concentrations of samples were added to each well, followed by the addition of an equal amount of diluted bacterial solution (1 × 10^6^ CFU/mL) per well, and the plate was incubated in a shaker (120 r/min, 37 °C) for 12 h. The plate was removed every 2 h and measured using an Enzyme Marker. The OD600 value was measured continuously for 12 h [52].

### 4.5. Investigation of the Antibacterial Mechanism

#### 4.5.1. Bio-SEM

The morphological changes of bacteria before and after treatment with sample were observed using SEM. A certain concentration of *S. aureus* and *E. coli* (1.0 × 10^8^ CFU/mL) was mixed with sample and co-cultured at 37 °C for 3 h. The supernatant was discarded after centrifugation (3000 r/min, 5 min), rinsed three times with PBS, resuspended by adding 2.5% glutaraldehyde and stored at 4 °C overnight. Fixation of bacterial shape was performed. The bacteria were dehydrated with 30%, 50%, 70%, 90% and 100% ethanol and observed by SEM [54].

#### 4.5.2. Determination of the ROS

The determination of ROS in bacterial cells was performed using the ROS kit from Nanjing Jiancheng [56]. Institute of Biological Engineering. First, certain concentrations of *S. aureus* and *E. coli* (1.0 × 10^8^ CFU/mL) were mixed with sample and co-cultured at 37 °C for 3 h. Bacterial bodies were collected by centrifugation (3000 r/min, 5 min), washed 3 times with PBS (1000 r/min. 5 min), then added 1 mL of 10μM of 2’,7’-dichlorofluorescein diacetate (DCFH-DA) and co-incubate for 1 h at 37 °C protected from light. After incubation, centrifuge (3000 r/min, 5 min), wash 3 times with PBS to wash off excess DCFH-DA, and add PBS to dilute the bacteria to the initial volume. The fluorescence intensity of the bacterial suspension was measured using a Hitachi F-7000 fluorescence spectrophotometer with excitation and emission wavelengths of 488 nm and 525 nm, respectively. Similarly, in the NAC treatment, 25 mM of NAC was added to the bacterial suspension together with the sample and incubated for 3 h at 37 °C. ROS measurements were performed as described above.

#### 4.5.3. Bacterial Protein Leakage

Bacterial protein leakage was determined using the BCA protein concentration kit from Beyoncé [59]. First, certain concentrations of *S. aureus* and *E. coli* (1.0 × 10^8^ CFU/mL) were mixed with sample and co-cultured at 37 °C for 3 h. The supernatant was collected by centrifugation (10,000 r/min, 10 min), followed by immediate incubation of the supernatant extract at −20 °C under freezing. The OD562 values were determined using an enzyme marker.

#### 4.5.4. Lipid Peroxidation Levels

Lipid peroxidation levels were measured using Beyoncé’s Lipid Peroxidation MDA kit. Certain concentrations of *S. aureus* and *E. coli* (1.0 × 10^8^ CFU/mL) were mixed with sample and co-cultured at 37 °C for 3 h. Bacterial bodies were collected by centrifugation (10,000 r/min, 5 min) and washed 3 times with PBS. Then, 300 μL Western cell lysate was added and centrifuged at 10,000 rpm for 10 min to remove the undissolved material. A total of 200 μL of cell lysate was mixed with 400 μL of thiobarbituric acid (TBA) reagent in a boiling water bath at 100 °C for 15 min. The water bath was cooled to room temperature and then centrifuged at 1000 rpm for 10 min. Then, 200 μL of supernatant was added to a 96-well plate and the absorbance was measured at 532 nm using an enzyme marker to obtain the lipid peroxidation level [59].

### 4.6. Cytotoxicity Test

The trypsin-digested AD293 or A549 cells were inoculated with 100 μL at a density of 1 × 10^5^ cell/well in a 96-well cell culture plate, and then incubated in a cell culture incubator (37 °C, 5% CO_2_). When the cells at the bottom of the plate grew to a density of 80–90%, the culture medium was discarded, rinsed carefully with PBS twice, 100 μL of sample/well at different concentrations was added, and normal cell control wells were set, and the culture was continued at 37 °C with 5% CO_2_. After 12 h of incubation, 20 μL of 5 mg/mL MTT was added to each well and the incubation was continued for 4 h. At the end of incubation, the cell supernatant was carefully discarded, 150 μL of DMSO was added to each well, and then the well plates were placed on a shaker and shaken uniformly for 10 min to allow the bottom metsan crystals to fully melt. Finally, the absorbance of each well at 492 nm was measured using an enzyme marker [52]. The cell viability was calculated according to the following equation:

Cell viability (%) = (OD samples − OD blank)/(OD control − OD blank) × 100%(3)

### 4.7. Hemolytic Assay

To further evaluate the biocompatibility of MIL-101(Fe)@ZnO, we determined its hemolysis rate. Chicken erythrocytes were collected by centrifugation at 1500 rpm for 10 min, the supernatant was discarded, and washed with PBS until the red color of the supernatant disappeared and it was no longer turbid. After centrifugation, 0.2 mL of the precipitate was added to 9.8 mL of PBS for resuspension, i.e., 2% chicken erythrocyte suspension was prepared. Different concentrations of samples and equal amounts of erythrocyte suspension were added to 1.5 mL EP tubes and then incubated at 37 °C for 2 h. PBS was used as the negative control group and 1% TritonX-100 as the positive control group. After incubation, the supernatant was centrifuged at 3000 rpm for 5 min, and 150 μL of the supernatant corresponding to the concentration was transferred to a 96-well plate with three replicate wells in parallel. The OD value at 540 nm was detected by enzyme marker. The percentage of hemolytic index (%) was calculated by using the following formula [61]:

Hemolysis (%) = (OD test samples − OD negative control)/(OD positive control − OD negative control) × 100%(4)

### 4.8. Statistical Analysis

All of the experiments were performed in triplicate. The data are expressed as the mean ± standard deviation. Statistical significance was assessed using Student’s *t*-test, and the values were considered to be significant at *p* < 0.05.

## 5. Conclusions

A new antibacterial MOF material (MIL-101(Fe)@ZnO(1:0.5)) was synthesized based on Zn^2+^ and MIL-101(Fe), and its structure was fully characterized. MIL-101(Fe)@ZnO(1:0.5) showed good inhibition performance against six hospital-susceptible bacteria.

Remarkably, the advantage of zinc-containing antimicrobial materials over traditional organic antimicrobial materials that eliminate bacteria through chemical mechanisms is that they act antimicrobially by physically and mechanically destroying bacterial cells or releasing ROS. These new materials play an important role in a non-toxic and safe antimicrobial approach, replacing some of the traditional antimicrobial agents such as organic disinfectants, antiseptics and antibiotics to mitigate the ecological impact.

Finally, as further progress in the development of Zn-containing antimicrobial materials will help realize more promising solutions for conventional disinfection and environmental decontamination, it is expected that MIL-101(Fe)@ZnO(1:0.5) will be used in antimicrobial coatings, drug control and other applications.

## Data Availability

Not applicable.
